# Regulation of Wnt receptor activity: Implications for therapeutic development in colon cancer

**DOI:** 10.1016/j.jbc.2021.100782

**Published:** 2021-05-14

**Authors:** Zhendong A. Zhong, Megan N. Michalski, Payton D. Stevens, Emily A. Sall, Bart O. Williams

**Affiliations:** Department of Cell Biology, Van Andel Institute, Grand Rapids, Michigan, USA

**Keywords:** Wnt, Lrp5, Lrp6, frizzled, Rnf43, Znrf3, colorectal cancer, APC, B-catenin, porcupine, APC, adenomatous polyposis coli, CK1, casein kinase 1, CRC, colorectal cancer, DVL, Dishevelled, GSK3, glycogen synthase kinase 3, LRP5, low-density lipoprotein receptor-related proteins 5, LRP6, low-density lipoprotein receptor-related proteins 6, PORCN, Porcupine, RNF43, ring finger protein 43, ZNRF3, zing and ring finger 3

## Abstract

Hyperactivation of Wnt/β-catenin (canonical) signaling in colorectal cancers (CRCs) was identified in the 1990s. Most CRC patients have mutations in genes that encode components of the Wnt pathway. Inactivating mutations in the adenomatous polyposis coli (*APC*) gene, which encodes a protein necessary for β-catenin degradation, are by far the most prevalent. Other Wnt signaling components are mutated in a smaller proportion of CRCs; these include a FZD-specific ubiquitin E3 ligase known as ring finger protein 43 that removes FZDs from the cell membrane. Our understanding of the genetic and epigenetic landscape of CRC has grown exponentially because of contributions from high-throughput sequencing projects such as The Cancer Genome Atlas. Despite this, no Wnt modulators have been successfully developed for CRC-targeted therapies. In this review, we will focus on the Wnt receptor complex, and speculate on recent discoveries about ring finger protein 43regulating Wnt receptors in CRCs. We then review the current debate on a new APC–Wnt receptor interaction model with therapeutic implications.

Colorectal cancer (CRC) is the second most common cause of cancer-related death and the third most prevalent malignant tumor worldwide. The American Cancer Society estimated that there would be some 150,000 new CRC cases and about 53,200 CRC-related deaths in the United States in 2020 (https://www.cancer.org/cancer/colon-rectal-cancer/about/key-statistics.html). Overall survival for CRC patients has steadily increased over the past several decades, mainly because of the success rates of surgical intervention. However, once tumors start to invade local tissues or metastasize to distant sites, 5-year survival rates drop to 71% and 14%, respectively (1, 2). The current treatment regimen for patients with metastatic disease includes various fluorouracil-based treatments, all of which have similar patient outcomes (3, 4, 5, 6, 7). Several targeted therapies have been approved over the past few years, including monoclonal anti-EGFR antibodies (cetuximab and panitumumab), anti-angiogenic therapies (bevacizumab and aflibercept), and BRAF inhibitors (reviewed in (8)). In addition, sequencing the genomic DNA of patient tumors has allowed these inhibitors to be more successfully used in some CRC patients. The recent approval of checkpoint inhibitors against CRC has opened the door to using previously approved chemotherapeutics combined with immunotherapies. However, these results are still unsatisfying regarding metastatic CRC patient outcomes, and future research should continue to focus on novel targeted therapies.

A major therapeutic target in CRC is the frequently mutated and activated Wnt signaling pathway. Hyperactivation of canonical Wnt signaling in CRC is often associated with the mutations inactivating the adenomatous polyposis coli (*APC*) gene. Mutations in other Wnt signaling pathway components are also associated with CRCs, although at a lower frequency. Anticancer drugs that target proteins throughout the Wnt signaling cascade are in preclinical and clinical studies, including CRC trials, but no Wnt-targeting therapies are part of current clinical practice. The lack of such therapeutics is partly because of an ongoing debate regarding receptor specificity and general toxicity caused by Wnt inhibition. This review will summarize this debate and discuss recent advances in Wnt receptor research that may inform new targeted therapies for CRC patients.

## Wnt/β-catenin signaling

WNTs are a family of 19 secreted glycolipoproteins in mammals that can activate Wnt signaling synergistically (9). Wnt signaling can broadly be classified as canonical (β-catenin-dependent) or noncanonical (β-catenin-independent). These pathways preferentially use different receptors and coreceptors to activate downstream signaling cascades, but this demarcation is not absolute. Some WNTs can engage multiple Wnt signaling pathways, depending on receptor context (10, 11, 12, 13, 14). The downstream canonical and noncanonical signaling cascades share some common intracellular components (15). Although we focus on canonical Wnt signaling in this review, noncanonical Wnt signaling is likely also affected when these common Wnt pathway components are manipulated.

**Figure 1:**
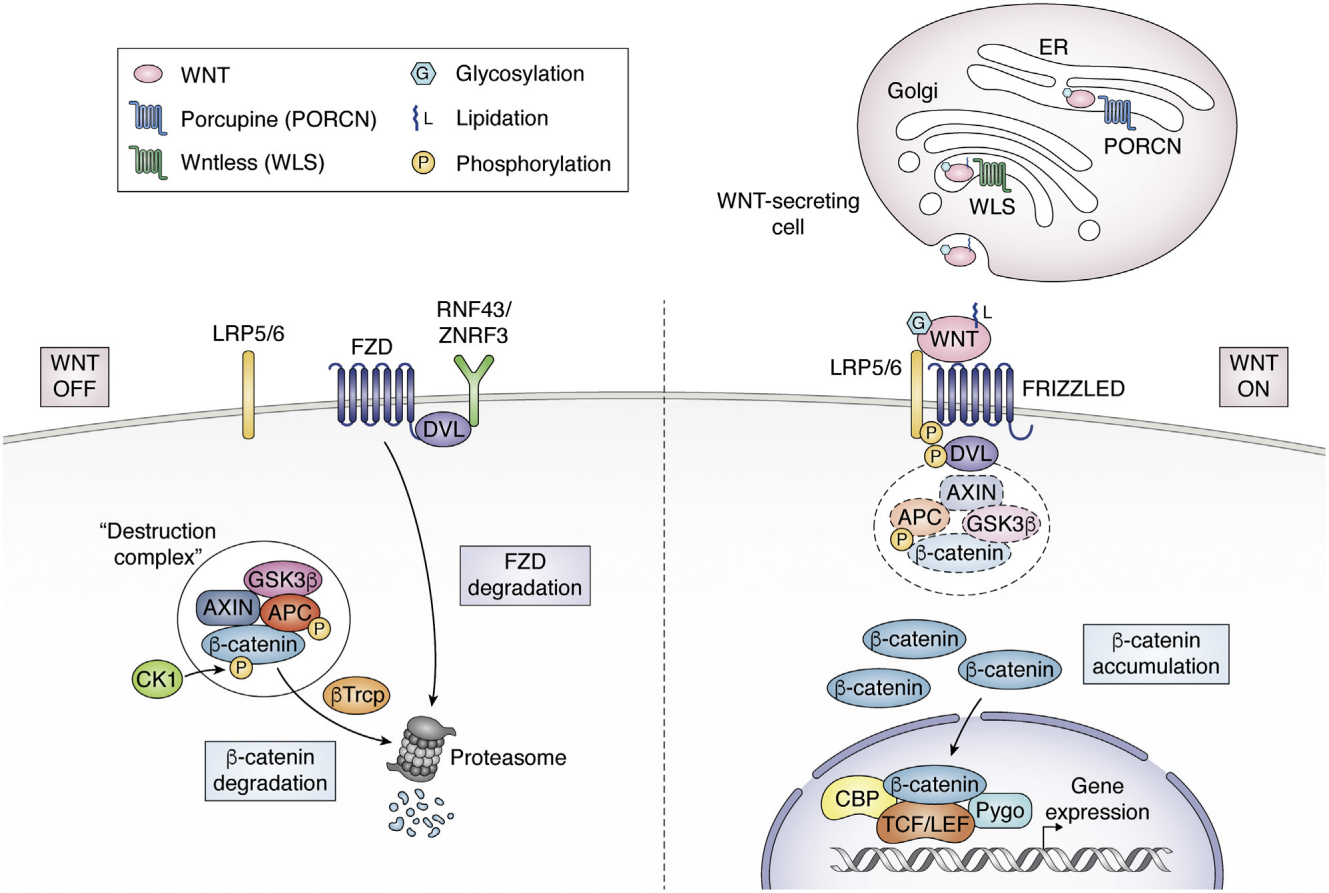
**Overview of the canonical Wnt/β-catenin signaling pathway.***Top*, WNT is lipid modified by PORCN in the endoplasmic reticulum (ER) and transported to the Golgi complex, where WLS palmitoleoylates and chaperones WNT to the plasma membrane. *Left*, β-catenin signaling is inhibited without WNT present (“WNT OFF”). FZD is removed from the cell membrane by RNF43/ZNRF3 binding through DVL. The β-catenin concentration is kept low by an intracellular multiprotein destruction complex that includes APC, axin, CK1, and glycogen synthase kinase 3 (GSK3), which facilitates the GSK3-dependent phosphorylation of β-catenin that targets it for ubiquitin-dependent proteolysis. This continuous degradation is vital in preventing the accumulation and nuclear translocation of β-catenin. *Right*, WNT causes the accumulation of β-catenin and activates signaling (“WNT ON”). When a WNT ligand engages the receptor complex, the C terminus of LRP5/6 is phosphorylated, creating a binding site for axin, resulting in inhibition of the destruction complex and GSK3 activity, allowing cytoplasmic levels of *β*-catenin to increase. *β*-Catenin subsequently translocates into the nucleus and complexes with LEF/TCF proteins and other co-factors to activate transcription of target genes. CK1, casein kinase 1; DVL, Dishevelled; LRP5, low-density lipoprotein receptor-related proteins 5; LRP6, low-density lipoprotein receptor-related proteins 6; RNF43, Ring finger protein 43; ZNRF3, Zing and ring finger 3.

WNT ligand–receptor interactions are complex, and the hydrophobic nature of WNTs has complicated attempts to understand these interactions in more detail. While the first *Wnt* gene was identified in 1982 (16), it took another 14 years to identify a cognate receptor (17). The purification of biologically active WNT protein was not reported until 2003 (18), and the first crystal structure of a WNT in complex with one of its receptors, frizzled 8, was solved in 2012 (19). These advances have helped us understand why the biochemistry associated with WNTs is so challenging. For example, WNTs contain 12 disulfide bonds. Also, a palmitoleic acid modification is required for WNT protein secretion (Fig. 1) and receptor binding (20), and this modification increases the hydrophobicity of the protein and makes it harder to work with *in vitro*.

Two proteins, Porcupine (PORCN) and Wntless, necessary for the proper secretion of WNT ligands, are located within the endoplasmic reticulum. PORCN first adds lipid modifications to glycosylated WNTs, which are essential for Wntless to bind and transport WNTs to the cell surface and out of WNT-secreting cells (Fig. 1) (20, 21, 22). During active Wnt/β-catenin signaling, WNTs bind to members of the frizzled (FZD) receptors and LRP5 or LRP6 (low-density lipoprotein receptor-related proteins 5 or 6) on WNT-receiving cells. WNT binding induces these receptors to assemble into large signalosome complexes. These complexes stimulate the recruitment of an intracellular protein, Dishevelled (DVL), which subsequently recruits the destruction complex to the ligand-bound, activated receptors. LRPs then undergo further phosphorylation leading to glycogen synthase kinase 3 (GSK3) inactivation and β-catenin stabilization (23, 24).

Ring finger protein 43 (RNF43) and zing and ring finger 3 (ZNRF3) are direct Wnt targets and negative feedback regulators of Wnt signaling (Fig. 1). RNF43 and ZNRF3 are homologous transmembrane ubiquitin ligases that specialize in removing frizzled (FZD) receptors from the cell surface by targeting them for ubiquitin-mediated endocytosis and lysosomal degradation (25, 26). DVL is involved in RNF43/ZRNF3–mediated FZD removal and also in active Wnt signaling (25). R-spondins (RSPO1-4), important stem cell factors of the intestines, can directly bind to RNF43/ZNRF3 and facilitate their removal from the cell membrane, thus increasing membranous FZDs and sensitizes cells to respond to WNT (27).

## Wnt/β-catenin signaling in CRC

**Figure 2:**
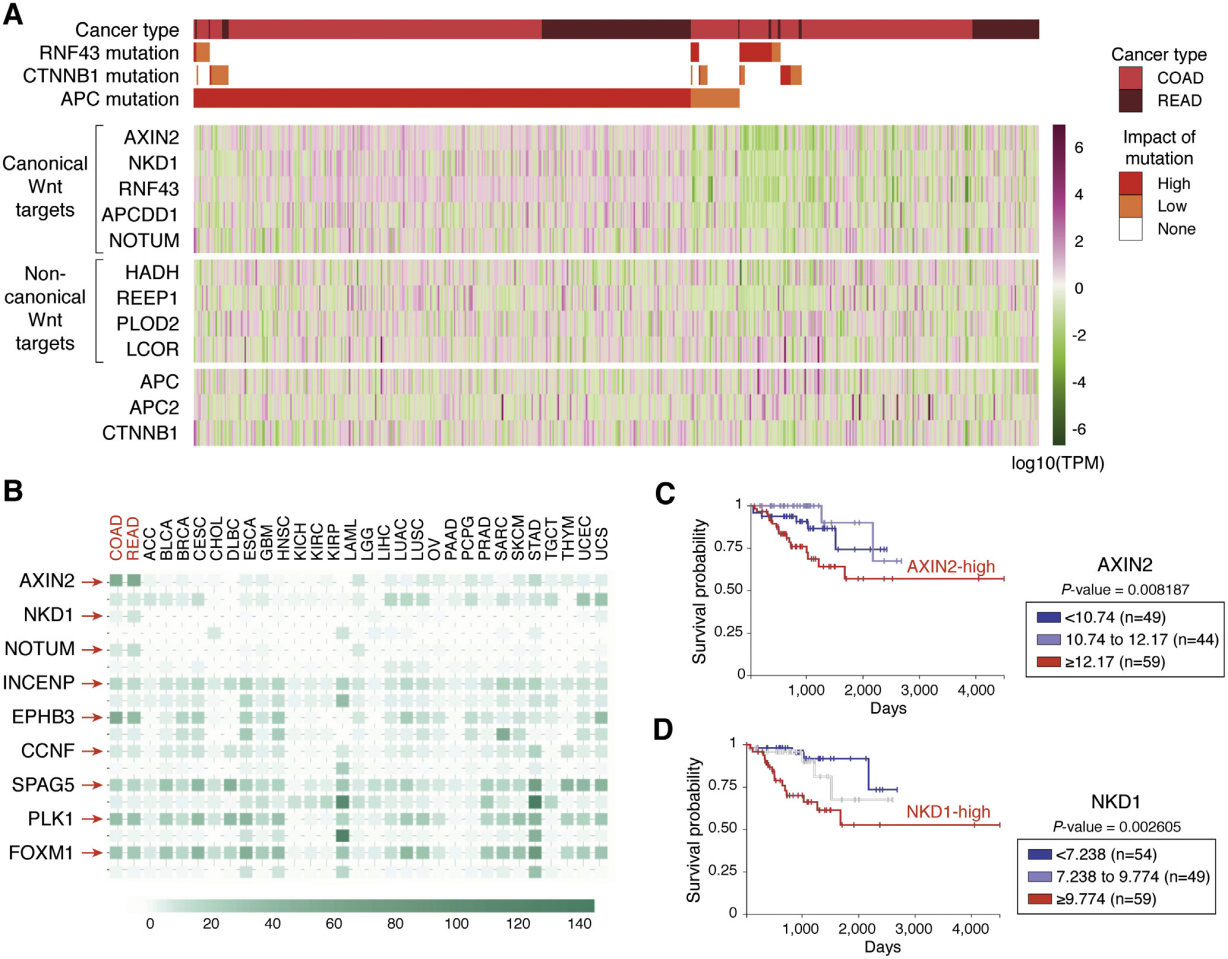
**Canonical Wnt signaling in CRC is associated with *APC*/*CTNNB1* mutations and patient outcomes.***A*, key *WNT* gene mutations in CRC are associated with canonical Wnt signaling. CRC samples (634 in total) from TCGA with both RNA sequencing and exome sequencing data available were analyzed for single nucleotide variations (SNV) in *APC*, *RNF43*, and *CTNNB1* as well as for gene expression (log10 of Transcripts Per Kilobase Million, TPM) of the indicated Wnt target genes and the *APC*/*APC2*/*CTNNB1* genes. *RNF43* itself is also a strong Wnt target gene. The heatmap shows Wnt/β-catenin activity represented by some of the most sensitive Wnt targets (*AXIN2*, *NKD1*, *RNF43*, *APCDD1*, *NOTUM*) identified by two studies using cancer cell lines (33, 37). Another study also identified these genes as leading-edge negative regulator genes to differentiate ligand-dependent and ligand-independent CRCs (28). As expected, the transcription of these Wnt target genes is significantly higher in CRCs with high-impact mutations in *APC* and *CTNNB1*. The newly proposed noncanonical Wnt target genes (*HADH*, *REEP1*, *PLOD2*, and *LCOR*) are shown (37). The mutations in APC having “high” impact on protein function are generally those that cause a complete deletion of the “mutation cluster region”. The mutations in *CTNNB1* having “high” impact are those with missense mutations within GSK3 phosphorylation sites. The mutations in *RNF43* having “high” impact are nonsense or frameshift mutations. *B*, a heatmap showing several sensitive Wnt targeting genes that are highly expressed in COAD or READ tumors (the first two columns). This pattern was not observed in other cancer types. The RNAseq data from matched TCGA tumor samples and GTEx normal samples are compared using the Gene Expression Profiling Interactive Analysis (GEPIA, http://gepia.cancer-pku.cn/). The *red arrows* indicate tumor samples of each cancer type. *C* and *D*, the association between expression of Wnt target genes *AXIN2* or *NKD1* and patient prolonged disease-free interval was evaluated using UCSC Xena Functional Genomics Explorer (https://xenabrowser.net/heatmap/). APC, adenomatous polyposis coli; COAD, colon adenocarcinoma; CRC, colorectal cancer; GSK3, glycogen synthase kinase 3; READ, rectal adenocarcinoma; TCGA, The Cancer Genome Atlas.

The majority of sporadic CRCs contain at least one alteration in known Wnt regulator genes, and mutations in *APC* are by far the most frequent. *RNF43* and β-catenin (*CTNNB1*) mutations are also present in some CRC patients but at a lower frequency. The mutations in either the *APC* or *RNF43* gene cause loss of function. Mutations in *CTNNB1* are gain of function: they typically encode forms of β-catenin that are resistant to GSK3-mediated phosphorylation and subsequent ubiquitin-mediated degradation. Mutations in *APC*, *CTNNB1*, and *RNF43* are virtually mutually exclusive in CRC (Fig. 2*A*) (28), consistent with them being part of the same signaling pathway. However, some CRC samples that lack mutations in *APC* or *CTNNB1* still have elevated Wnt/β-catenin activity. This could be because of mutations in other components that can directly or indirectly impact Wnt signaling or epigenetic mechanisms (29). For example, recent studies identified the Wnt signaling pathway as a major target of P53 in murine systems that regulate stem cell differentiation and cancer metastasis (30, 31, 32).

Based on mRNA analysis of a set of classic Wnt targets and detection of nuclear β-catenin protein levels in cancer tissues, β-catenin signaling is elevated in ligand-independent CRC samples that had either *APC* or *CTNNB1* mutations, (28, 29). In fact, colon adenocarcinoma and rectal adenocarcinoma present the most dramatic increase in Wnt/β-catenin target genes across all The Cancer Genome Atlas tumors (Fig. 2*B*) (33). High expression of AXIN2 and NKD1 (NKD Inhibitor Of Wnt Signaling Pathway 1, NAKED-1) have a strong negative association with a prolonged disease-free interval among both colon adenocarcinoma and rectal adenocarcinoma patients (Fig. 2, *C* and *D*), implying that the inhibition of Wnt/β-catenin signaling in CRC patients could help to reduce relapse after treatment. It is also possible that tumor-intrinsic active β-catenin signaling could result in T-cell exclusion and resistance to immunotherapies (34, 35, 36).

Because Wnt signaling regulates cell growth and differentiation, it is not surprising that its dysregulation is commonly associated with tumorigenesis. However, despite substantial efforts, no therapies that target the pathway have been clinically effective. This is not only because of technical challenges and/or the side-effects associated with targeting components of the pathway but also because of the complex regulation of the pathway by many Wnt components. Nonetheless, stabilization of β-catenin, the hallmark of canonical Wnt activation, results in the tumorigenic phenotypes often observed in CRC. So, the stabilized β-catenin remains the most promising target for curing the disease. In the following sections, we will focus on recent discoveries related to RNF43 and APC in CRC and the therapeutic implications of these discoveries.

## Blocking Wnt secretion to treat RNF43-mutant CRC

**Figure 3:**
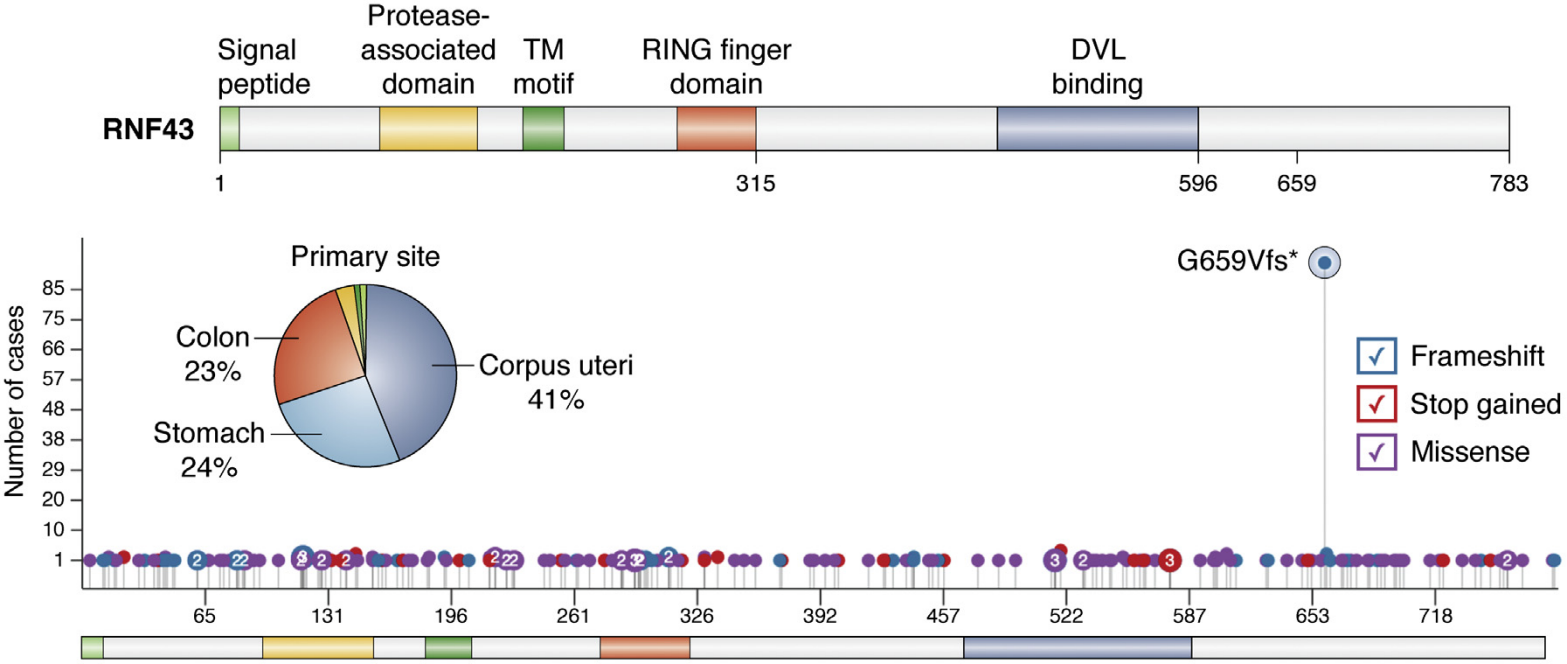
**RNF43 mutations in human cancers.***Top*, human RNF43 protein domains. The numbers indicate amino acid numbers of the human RNF43 protein. *Bottom*, the *dot colors* indicate the mutation types, and the Y-axis shows the number of cases with mutations across RNF43 protein domains (X-axis). The *pie chart* shows the number of cases of TCGA primary cancers having RNF43 mutations. Data from Genomic Data Commons Data Portal (NIH). DVL, Dishevelled; RING, RING finger domain; TCGA, The Cancer Genome Atlas; TM, transmembrane motif.

RNF43 and ZNRF3 are major Wnt antagonists and Wnt/β-catenin downstream targets (25, 26), making them important negative feedback regulators for the Wnt signaling pathway. RNF43 loss-of-function mutations can presumably lead to Wnt signaling activation by increasing FZD receptors on the cell surface, sensitizing cells to WNTs. RNF43 is the third most highly mutated Wnt gene in CRCs, which is in line with RNF43 (but not ZNRF3) being highly expressed in the intestine (27, 38). RNF43 mutation is also common in stomach, corpus uteri, and other cancers, one of which is the highly recurrent *RNF43* frame-shift mutation, G659Vfs (Fig. 3).The loss of RNF43 protein expression is significantly more common in colorectal and gastric tumor samples with G659Vfs than in those with WT RNF43 (39). Single-cell RNA-sequencing data from the Tabula Muris project pinpoints the large intestine epithelial cell as the highest Rnf43-expressing cell type among 20 adult mouse organs (40). These observations suggest that RNF43 mutations could contribute to CRC pathogenesis.

Some studies have aimed to reduce tumorigenesis in models of RNF43 loss-of-function by targeting WNT secretion. PORCN is a WNT-specific *O*-acyltransferase (Fig. 1), without which all WNT secretion from cells is blocked. Mutations in PORCN have been linked to several human diseases in which Wnt signaling is affected during embryogenesis and development (41). Blocking WNT secretion by using PORCN inhibitors such as LGK974 and C59 has been highly efficient in inhibiting Wnt/β-catenin signaling *in vitro* and *in vivo*. In a mouse colon cancer model with intestinal epithelium-specific deletion of both *Rnf43* and *Znrf3*, tumorigenic growth was strongly inhibited by C59 treatments. In contrast, the adjacent normal intestinal epithelium was not affected (42), which indicated WNT blockage was well-tolerated in normal tissues. Another study showed that cancer cell lines with inactivating *RNF43* mutations could be growth-inhibited with PORCN inhibitors *in vitro* and *in vivo* (43). Wnt secretion blocking is also very effective in inhibiting CRC growth using patient-derived xenograft models with RSPO3 fusion proteins that potentially engage RNF43/ZNRF3 to increase membrane FZD levels and sensitize Wnt response (44). This evidence proves that RNF43/ZNRF3 loss of function in cancer cells is still dependent on Wnt ligand stimulation. So, blocking local WNT secretion is a promising way to suppress RNF43-mutant tumors. There are current clinical trials for patients with BRAF-mutant metastatic CRC that use the Novartis PORCN inhibitor LGK974, either in combination with an immunomodulator (NCT01351103) or with the BRAF/EGFR inhibitors (NCT02278133).

One caveat of using PORCN inhibitors is that highly efficient blocking of Wnt secretion is required to inhibit Wnt signaling. Most *in vitro* and *in vivo* studies using PORCN inhibitors have adopted significantly higher concentrations than their labeled IC_50_ values to block WNT secretion effectively and stop tumor cell growth. Fortunately, the intestine, one of the most sensitive organs to Wnt signaling, can tolerate high (pharmacological level that can effectively inhibit grafted tumor growth) exposure to C59 without showing any phenotypic changes, perhaps because of the expression of drug efflux pumps in Wnt-producing cells (45, 46). However, when a higher amount of C59 was used or the Porcupine gene was deleted in the gut, severe intestinal homeostasis impairment was observed due to intestine stromal WNT blockage (46, 47).

Unexpectedly, some of the frequent *RNF43* mutations found in CRC patients, including the most common RNF43-G659Vfs, actually retain the ability to eliminate FZDs from the cell membrane and attenuate Wnt/β-catenin signaling (at least in a FZD overexpression cell culture system) (48, 49). This brings up the question of whether these RNF43 mutations confer WNT dependence onto these cancers. In line with these findings, *RNF43* mutations in COREAD samples are not associated with higher Wnt/β-catenin targets (Fig. 2*A*). In fact, the signet-ring cell carcinoma, a rare subtype of CRC with a high (34.5%) *RNF43* mutation rate, actually showed significantly inhibited Wnt/β-catenin signaling than those without *RNF43* mutations (50). One possibility is that the *RNF43* mutants found in CRC patients may play a role in regulating noncanonical Wnt signaling, which has many downstream pathways and targets. One study showed that some point mutations at the N terminus (around the PA domain) of RNF43 could switch it from a Wnt antagonist to a Wnt agonist or may disable RNF43 from suppressing canonical Wnt/β-catenin signaling while leaving its noncanonical Wnt inhibition role intact. However, the noncanonical Wnt activation outcome in this study was limited to DVL2 phosphorylation (51), and some newly proposed noncanonical Wnt target genes are not transcriptionally elevated in *RNF43*-mutant CRC samples (Fig. 2*A*) (37). Interestingly, recent studies suggest that mutant forms of RNF43 may be doing more than regulating FZD dynamics on the cell membrane. Some truncating mutations at the C-terminus of RNF43 robustly activate β-catenin signaling by trapping casein kinase 1 (CK1) at the cell membrane, thus preventing β-catenin degradation (52). Further investigation on the effects of *RNF43* mutations on noncanonical Wnt signaling regulation is necessary.

In summary, the presence of *RNF43* mutations in CRCs and the effectiveness of PORCN inhibitors on a number of RNF43-mediated CRC models provide optimism and opportunity to develop therapeutics targeting Wnt signaling. However, caution is warranted when interpreting the effects of *RNF43* mutations, because possibly not all involve loss of function. The impact of *RNF43* mutations on noncanonical Wnt signaling also needs to be carefully studied regarding its impact on tumor growth and metastasis. While new efforts are addressing how *RNF43* mutations affect Wnt receptor availability and downstream signaling, much work has already been done on how *APC* mutations regulate CRC initiation and progression. Nonetheless, a novel model involving APC’s interaction with the Wnt receptor complex has been proposed and debated in the field.

## Targeting Wnt receptors to inhibit β-catenin in APC-mutant cells

APC is a multifunctional and crucial tumor suppressor gene in the gastrointestinal tract, and mutations causing APC inactivation are found in about 80% of all human colon tumors. Loss-of-function mutations in one allele of APC are widely recognized as the initiating event (“first hit”) in most CRC cases (both sporadic and familial) as well as in CRC animal models. Familial adenomatous polyposis patients with heterozygous germline APC mutations can develop hundreds of colon tumors that are precancerous. Their removal is recommended to avoid progression to invasive and metastatic tumors (53). Murine models and other model animals have confirmed that heterozygosity of APC mutations similar to those seen in CRC patients produce an autosomal dominant colon cancer predisposition (54). In both mouse models and familial adenomatous polyposis patients with heterozygous APC mutations, mutation of the other WT APC allele (“second hit”) is associated with tumorigenesis.

### APC loss of function is different from β-catenin gain of function

Why do mutations in APC result in aggressive colorectal cancer when compared with mutations which directly activate β-catenin? We have long known that human colon cancers having β-catenin mutations grow less aggressively than those with APC mutations (55). We also know that colorectal tumors with APC loss of function are transcriptionally distinct from colorectal tumors with hyperactivated Wnt signaling caused by other non-APC–mediated mechanisms (56). The APC protein contains both nuclear localization signals and nuclear export signals that enable its shuttling between the nucleus and cytoplasm. While in the nucleus, APC can interact with β-catenin, which results in transcriptional repression of Wnt target genes. Most APC mutations found in CRC patients can both reduce APC protein levels and prevent β-catenin from exiting the nucleus (57, 58, 59). Although it has not been confirmed that APC is directly involved in regulating gene transcription, an anti-APC ChIP-seq study showed that APC binding sites overlap with a large portion of the canonical Wnt transcription factor TCFL2/TCF4 and AP-1 binding sites. Thus, APC can regulate a wide range of the cellular processes that AP-1 is responsible for, including cell growth, differentiation, and apoptosis (60).

### APC2 is a homolog of APC

APC2 (APC regulator of WNT signaling pathway 2) is a mammalian APC homolog and closely resembles APC in its overall domain structure. Overexpression of either APC or APC2 in colon cancer cells that lack functional APC could inhibit Wnt signaling effectively (61, 62). Mammalian APC and APC2 are at least partially compensatory in specific tissues. For example, knocking out either Apc or Apc2 in the mammary gland fails to induce Wnt/β-catenin signaling or any phenotypic changes. In contrast, concomitant mammary *Apc* and *Apc2* deletion can cause squamous carcinoma with elevated β-catenin levels (63). In aged mice with a hypomorphic *Apc* allele, an Apc2 deficiency resulted in a predisposition to granulosa cell tumor formation and tumor-related Wnt/β-catenin signaling activation (64). Consistently, the *Apc*^*min/+*^ mammary epithelium did not show any epithelial lesions or reduced proliferation until lactation began, which is when APC expression reaches its peak. Interestingly, β-catenin signaling appears to be unchanged in *Apc*^*min/+*^ mammary tissue (65), suggesting that APC2 could completely compensate for APC in the mammary to keep ß-catenin signaling in check while participate in other roles such as maintaining epithelial integrity. Consistently, dAPC and dAPC2 function in a partially redundant manner in *Drosophila*, although these isoforms have distinct tissue distribution and subcellular localizations (66, 67, 68). Interestingly, the homozygous knockout of dAPC2 is embryonically lethal because of elevated Wg signaling in *Drosophila* (69), whereas *Apc2*-deficient mice are viable and virtually normal-looking, except for reduced female fertility as homozygotes and a mild increase in the number of intestinal cells expressing nuclear β-catenin (64, 70). Although APC2 expression is significantly decreased in CRC samples (71, 72) and associated with CRC patients (71), More work on APC2 for human cancers is needed.

## A new model of APC/APC2–LRP5/6 interaction

It had been assumed that the loss of APC led to β-catenin stabilization and activation of target genes regardless of upstream receptor status. This assumption was based on the absolute requirement for APC in the destruction complex that targets β-catenin for degradation. However, recent reports have suggested that upstream signals (such as FZD, DKK, sFRPs, and WNTs) can still affect Wnt signaling in APC-mutant colorectal and gastric cancer cell lines (73, 74, 75). This suggests that mutant or truncated APC proteins in CRC patients can participate in destruction complex formation or facilitate β-catenin ubiquitination (24). Even so, the destruction complex containing a mutant APC may be defective in responding to some upstream signaling, such as responding to a WNT cue to relocate the destruction complex toward the plasma membrane (76). Higher levels of Wnt expression in Apc mutant cells and their ability to remain responsive to these increased levels of ligand may also contribute to signaling (77).

**Figure 4:**
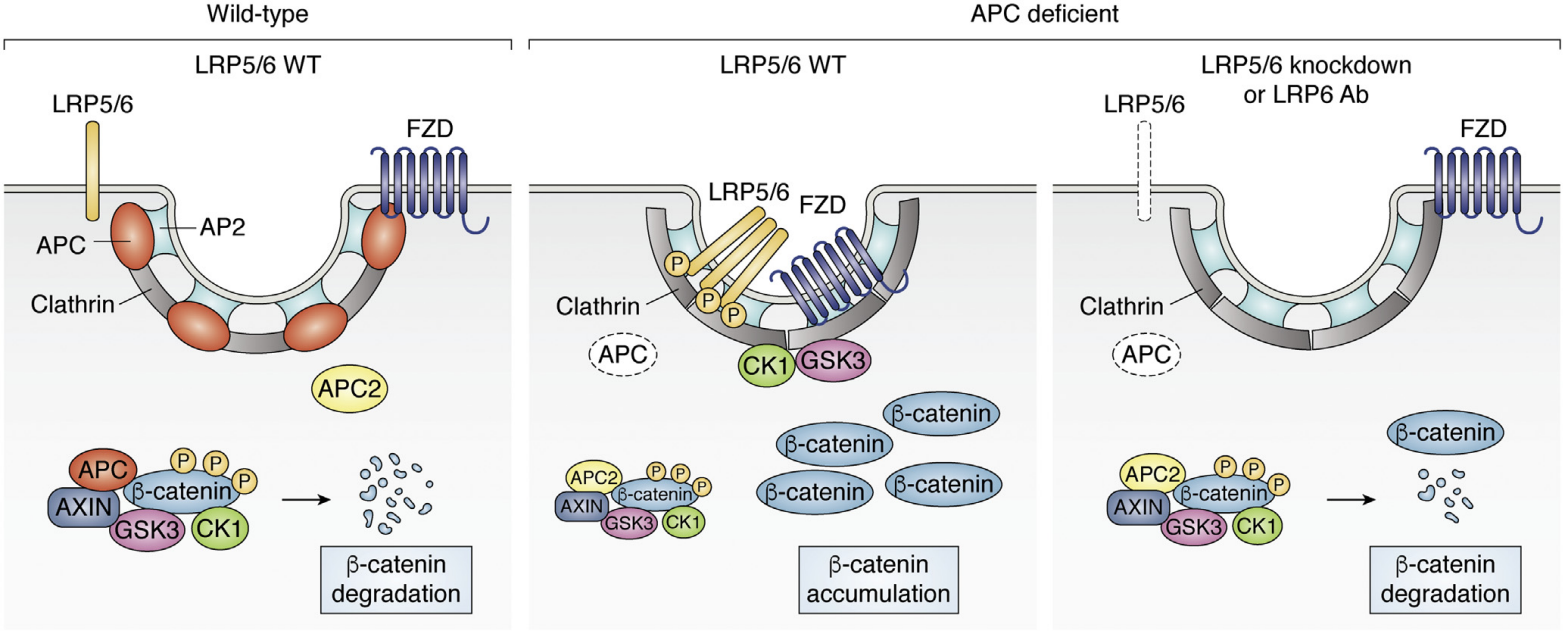
**APC-deficiency induces Wnt signaling through the clathrin endocytic pathway.***Left*, in wild-type cells, APC works with axin, GSK3, and CK1 to degrade β-catenin protein and binds to clathrin to suppress Wnt-independent signalosome assembly. *Middle*, when APC is knocked down (or mutated), APC2 cannot compensate for APC to suppress signalosome formation, which leads to β-catenin protein accumulation in the cell. *Right*, in the events of LRP knockdown or treatment with LRP6 antibody or with FZD or DVL antagonists, the signalosome is no longer active, and the APC2-mediated destruction complex continues to degrade β-catenin, which leads to much suppressed Wnt/β-catenin signaling (*right panel*). APC, adenomatous polyposis coli; CK1, casein kinase 1; DVL, Dishevelled; GSK3, glycogen synthase kinase 3; LRP6, low-density lipoprotein receptor-related proteins 6.

A novel and compelling signaling model has been proposed recently by Saito-Diaz *et al.* (78), in which APC plays a role in suppressing the ligand-independent Wnt signalosome (a multiprotein signaling platform consisting of LRP6, FZD, DVL, AXIN, CK1, GSK3β, and other intracellular signaling components) assembly by binding to clathrin. If APC is lost, APC2 can compensate in destruction complex formation but cannot replace the function of APC in signalosome suppression (see Fig. 4 (modified from (78)) for a more detailed description). One difference between APC and APC2 is that APC knockdown by siRNA can rapidly induce LRP6 phosphorylation (signalosome activation), even though knockdown of either APC or APC2 can increase β-catenin levels in HEK293 cells. The clathrin-interacting region in APC has not been identified and is presumably missing in both APC2 and C-terminus-truncated APC. In this new model, β-catenin stabilization after APC loss is dependent on both the disassembly of the APC/AXIN/GSK3/CK1-mediated destruction complex and the assembly of signalosome complexes (Fig. 4).

In other words, hyperactivated Wnt/β-catenin signaling in APC-mutant or APC-deficient cells could be dramatically reduced by knockdown or chemical inhibition of the receptor components (LRP, FZD, or DVL, but not WNTs) or with an LRP6 antibody (78). This was confirmed in *APC*^*min/+*^ organoids and *Drosophila*. Importantly, the translational implications from this study included targeting the Wnt receptor complex to inhibit Wnt hyperactivation caused by APC inactivation in cancers like CRC. For example, vantictumab (a pan-FZD antibody), which has been used in clinical trials to treat breast, pancreatic, and ovarian cancers, could be repurposed to treat APC-mutant CRC patients. However, a better strategy is needed to gain more specificity and avoid side-effects caused by a systematic WNT inhibition, such as detrimental effects on bone (79). One example is developing FZD5-specific antibodies, with an expectation that targeting only FZD5-expressing cells will not cause as severe systemic toxicity as porcupine inhibitors or pan-FZD antibodies will (80).

## Contradictory evidence on APC–LRP6 interaction

In a recent letter in *Developmental Cell* (81), Chen *et al.* argued that LRP5/6 were indeed not required for APC-deficiency–induced Wnt activation. This work was based on CRISPR/Cas9 technology using some of the same key cell lines used by Saito-Diaz *et al.* (78). Surprisingly, Chen found that LRP5/6 knockout in HEK293 T cells did not affect Wnt activation in responding to APC-deficiency. Chen further suggested that side-effects of the siRNA-mediated gene knockdown used in Saito-Diaz work might have contributed to the unexpected outcomes. For example, the LRP6 knockdown may have off-target effects specifically in APC-deficient HEK293 T cells and a number of APC-mutant colon cancer cell lines, and LRP6 knockdown in these cell lines further increased Wnt signaling instead of decreasing shown by Saito-Diaz (78). Although the mechanism of the off-target effects of LRP6 siRNAs in these cells was not clear, LRP6 knockdown did significantly reduce LRP6 protein and abolished Wnt3a response in wild-type HEK293 T cells, which verifies siRNA knockdown efficiency not being the discrepancy (81).

In another letter in the same issue, Cabel *et al.* (82) presented additional information by measuring β-catenin nuclear localization in single cells to confirm LRP’s involvement in responding to APC deficiency. They suggested that CRISPR/Cas9-mediated LRP5/6 knockout could result in compensation by genetic mechanisms that maintain elevated Wnt signaling over time in knockout cell clones. Although it has been reported that CRISPR-induced DNA double-strand breaks can induce a stress response (83), the cell stress response was reported to be uninheritable through cell divisions (84). It would be somewhat surprising if a compensatory genetic mechanism could block Wnt receptor signaling completely in APC-deficient cell clones. Still, we appreciate that the clonal variation can sometimes be significant enough to show a dramatic effect (85). The variation in responding to APC inactivation is also evidenced *in vivo* by the fact that Apc conditional knockouts in most mouse organs lead to dramatically induced Wnt/β-catenin signaling and cause significant phenotypes except in the mammary glands, which is at least partly because of compensation from Apc2 (63).

We may also consider cellular heterogeneity and differential compensatory signaling mechanisms (depending on the timing of gene deletions or knockdowns) as possible explanations for these differences. Some technical differences between the two studies could also be confounding factors. For example, APC protein truncation by CRISPR could lead to a gain-of-function APC mutant. At the same time, siRNA-mediated APC mRNA loss is probably not as complete as CRISPR-mediated APC truncation. The removal of the entire APC mRNA is not physiologically relevant to human colon cancer patients because most CRC patients retain APC N-terminal fragments. Finally, the levels of APC2 expression in specific cell types (or even subclones of cell lines) may influence the response to loss of APC.

The clathrin-mediated endocytic pathway was proposed to be required for Wnt activation in APC-deficient cells (78). However, a recent study reported that endocytosis (either clathrin- or caveolin-mediated) is not necessary for ligand-induced (but not APC-deficiency–induced) Wnt signaling (86). This study used genetically modified mouse embryonic stem cells and some other cell lines to observe β-catenin nuclear localization in live cells as the Wnt activation readout (86). This is quite a surprising finding because endocytosis has been widely recognized as important for Wnt signaling at different levels, such as endocytic trafficking in the WNT source cell and Wnt-receptor complex–induced signal activation (signalosome formation) (87). The underlying mechanisms of WNT-induced clathrin- or caveolin-mediated endocytosis are under debate (24, 88, 89, 90). Nevertheless, in cancer cells, components of the Wnt signaling and endocytosis pathways may be drastically different from those of normal cells. This endocytosis-independent β-catenin activation/nuclear localization is based on WNT recombinant protein treatment without the involvement of WNT-producing cells. It will be important to show whether APC-deficiency-induced Wnt signaling requires endocytosis using the same model (*i.e.*, nontumor cells). Additional effort is needed to define further the involvement of endocytosis in Wnt signaling under other contexts.

## Future directions

It is crucial to resolve these debates on the requirements for LRP5/6 in APC-deficiency–induced Wnt/β-catenin activation to pursue therapies optimally. The seemingly contradictory mechanisms reported could be because of cell-specific differences in other components of the Wnt pathway (such as APC2 expression level). Further examination of this question could identify a cellular signature that would allow informed predictions of whether specific APC-deficient CRC tumors would be more likely to respond to agents targeting the Wnt receptor complex.

Wnt signaling has been intensely studied for several decades. Just when we think that we have a complete understanding of its intricacies, unexpected observations emerge. In addition to the areas outlined above, there are still open questions about the stoichiometry of ligand-receptor complexes. In addition, the broad question of how specificity is generated for downstream signaling by the 19 Wnt ligands, ten Frizzled receptors, and the cohort of additional co-receptors is also of continuing interest. Clarifying these and other questions will undoubtedly identify new opportunities for therapeutic development and keep this research area vibrant for many years to come.

## Conflict of interest

The authors declare that they have no conflicts of interest with the contents of this article.
